# The genome sequence of the Copper-tipped Ermine, *Pseudoswammerdamia combinella* (Hübner, 1786) (Lepidoptera: Yponomeutidae)

**DOI:** 10.12688/wellcomeopenres.27367.1

**Published:** 2026-08-21

**Authors:** Laura Sivess, Gavin R. Broad, Stephanie Holt, Natural History Museum Genome Acquisition Lab, Darwin Tree of Life Barcoding Collective, Wellcome Sanger Institute Tree of Life Management, Samples and Laboratory team, Wellcome Sanger Institute Scientific Operations: Sequencing Operations, Wellcome Sanger Institute Tree of Life Core Informatics team, Tree of Life Core Informatics collective, Darwin Tree of Life Consortium

**Affiliations:** 1 Natural History Museum, London, England, UK

**Keywords:** Pseudoswammerdamia combinella, Copper-tipped Ermine, genome sequence, chromosomal, Lepidoptera

## Abstract

We present a genome assembly from an individual female *Pseudoswammerdamia combinella* (Copper-tipped Ermine; Arthropoda; Insecta; Lepidoptera; Yponomeutidae). The genome sequence has a total length of 604.80 megabases. Most of the assembly (99.84%) is scaffolded into 32 chromosomal pseudomolecules, including the W and Z sex chromosomes. The mitochondrial genome has also been assembled, with a length of 16.16 kilobases. This assembly was generated as part of the Darwin Tree of Life project, which produces genomes for eukaryotic species found in Britain and Ireland.

## Species taxonomy

Eukaryota; Opisthokonta; Metazoa; Eumetazoa; Bilateria; Protostomia; Ecdysozoa; Panarthropoda; Arthropoda; Mandibulata; Pancrustacea; Altocrustacea; Allotriocarida; Hexapoda; Insecta; Dicondylia; Pterygota; Neoptera; Eumetabola; Endopterygota; Aparaglossata; Panorpida; Amphiesmenoptera; Lepidoptera; Glossata; Neolepidoptera; Heteroneura; Ditrysia; Yponomeutoidea; Yponomeutidae; Yponomeutinae; *Pseudoswammerdamia*; *Pseudoswammerdamia combinella* ( Hübner, 1786) (NCBI:txid1870704).

## Background

*Pseudoswammerdamia combinella* ( Hübner, 1786), belonging to the family Yponomeutidae (subfamily Yponomeutinae), is sometimes known as either the Copper-tipped Ermel or the Little Buff-tip. It is a common species across Britain A review of the status of microlepidopte:contentReference [oaicite:0]index = 0, 2012 ( Davis, 2012), although found more sparsely in Scotland and Northern Ireland. Exclusively found in the Palaearctic region, *P. combinella* is found within Europe as far south as southern Italy (Calabria region) and as far north as Sweden (Svealand region) ( GBIF Secretariat, 2023).

Adults are on the wing between April and June and characterised by a relatively large wingspan, of 13–16 mm, pale colouration (ranging from sandy to grey) and a diagnostic orange spot at the end of the forewing, surrounded by a dark brown border, which separates them from species of *Paraswammerdamia* and *Swammerdamia* ( Sterling *et al.*, 2012). Larvae feed on Blackthorn ( *Prunus spinosa*), initially acting as leaf miners, before spinning a web and feeding beneath the leaves. Larvae pupate and overwinter in robust white cocoons on the ground ( Sterling *et al.*, 2012). Although a relatively small moth, *Pseudoswammerdamia combinella* has the distinction of bearing the longest scientific name of any UK moth!

**Figure 1.**
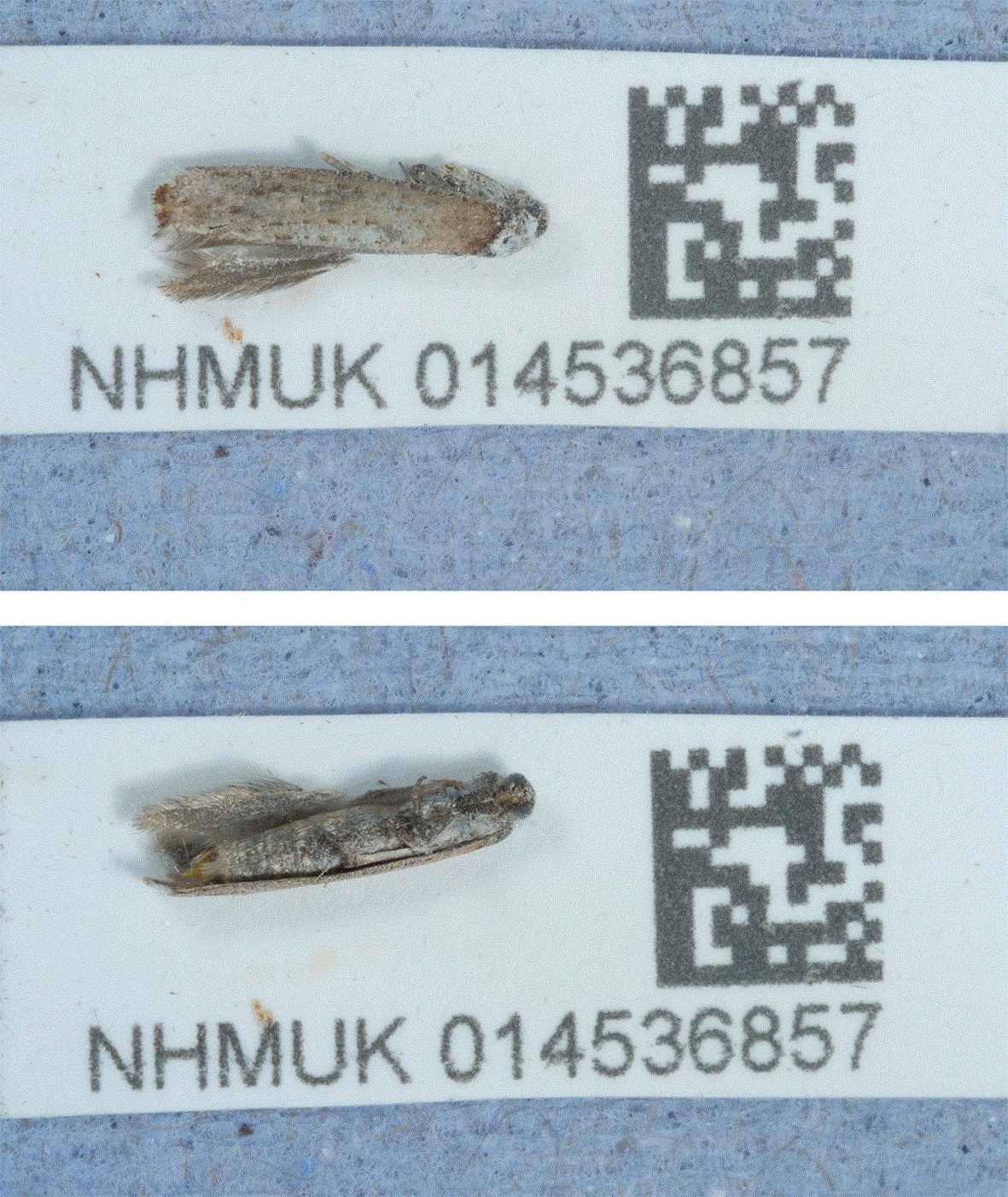
Photograph of the *Pseudoswammerdamia combinella* (ilPseComb1) specimen used for genome sequencing.

A genome sequence of *P. combinella* was produced as part of the Darwin Tree of Life project. The specimen used for sequencing ( Figure 1) was collected from Gilbert White’s House, Selborne, United Kingdom, during a targeted field sampling effort. This genome assembly supports comparative and functional genomic research into insect neurobiology, the development of the visual system, and specialisation on host plants. It also adds to the expanding collection of Lepidopteran genomic resources, supporting broader investigations into molecular evolution and adaptation across the order.

## Methods

### Sample acquisition and DNA barcoding

Two female *Pseudoswammerdamia combinella* specimens were used: the genome-sequencing specimen (specimen ID NHMUK014536857, ToLID ilPseComb1; Figure 1) and the Hi-C specimen (specimen ID NHMUK013697056, ToLID ilPseComb2). The genome-sequencing specimen was collected from Gilbert White’s House, Selborne, England, UK (latitude 51.09, longitude −0.94) on 2021-06-10 by Stephanie Holt, Gavin Broad, and Laura Sivess, and identified by Gavin Broad. The Hi-C specimen was collected from Bradenham, England, UK (latitude 51.68, longitude −0.81) on 2022-04-08 by David Lees, who also identified it.

The initial identification was verified by an additional DNA barcoding process according to the framework developed by Twyford *et al.* (2024). A small sample was dissected from the specimen and stored in ethanol, while the remaining parts were shipped on dry ice to the Wellcome Sanger Institute (WSI) (see the protocol). The tissue was lysed, the COI marker region was amplified by PCR, and amplicons were sequenced and compared to the BOLD database, confirming the species identification ( Crowley *et al.*, 2023). Following whole genome sequence generation, the relevant DNA barcode region was also used alongside the initial barcoding data for sample tracking at the WSI ( Twyford *et al.*, 2024). The standard operating procedures for Darwin Tree of Life barcoding are available on protocols.io.

### Nucleic acid extraction

Detailed protocols for nucleic acid extraction developed at the Wellcome Sanger Institute (WSI) Tree of Life Core Laboratory are available on protocols.io ( Howard *et al.*, 2025). The ilPseComb1 specimen was weighed and triaged to determine the appropriate extraction protocol. For high molecular weight (HMW) DNA extraction, 2.0 mg of tissue from the head, thorax and abdomen was used. The tissue was homogenised by powermashing using a PowerMasher II tissue disruptor. HMW DNA was extracted in the WSI Scientific Operations core using the Automated MagAttract v2 protocol. DNA was sheared into an average fragment size of 12–20 kb following the Megaruptor®3 for LI PacBio protocol. Sheared DNA was purified by manual SPRI (solid-phase reversible immobilisation). The concentration of the sheared and purified DNA was assessed using a Nanodrop spectrophotometer and Qubit Fluorometer using the Qubit dsDNA High Sensitivity Assay kit. Fragment size distribution was evaluated by running the sample on the FemtoPulse system. For this sample, the final post-shearing DNA had a Qubit concentration of 6.97 ng/μL and a yield of 313.65 ng, with a fragment size of 15.5 kb. The 260/280 spectrophotometric ratio was 2.49, and the 260/230 ratio was 1.88. The Genomic Quality Number (GQN) was 7.7.

### PacBio HiFi library preparation and sequencing

Library preparation and sequencing were performed at the WSI Scientific Operations core. Libraries were prepared using the SMRTbell Prep Kit 3.0 (Pacific Biosciences, California, USA), following the manufacturer’s instructions. The kit includes reagents for end repair/A-tailing, adapter ligation, post-ligation SMRTbell bead clean-up, and nuclease treatment. Size selection and clean-up were performed using diluted AMPure PB beads (Pacific Biosciences). DNA concentration was quantified using a Qubit Fluorometer v4.0 (ThermoFisher Scientific) and the Qubit 1X dsDNA HS assay kit. Final library fragment size was assessed with the Agilent Femto Pulse Automated Pulsed Field CE Instrument (Agilent Technologies) using the gDNA 55 kb BAC analysis kit.

The sample was sequenced using the Sequel IIe system (Pacific Biosciences, California, USA). The concentration of the library loaded onto the Sequel IIe was in the range 40–135 pM. SMRT Link software, a PacBio web-based workflow manager, was used to set up and monitor the run, and to perform primary and secondary analysis of the data upon completion.

### Hi-C

***Sample preparation and crosslinking***

The Hi-C sample was prepared from 20–50 mg of frozen head and thorax tissue from the ilPseComb2 sample using the Arima-HiC v2 kit (Arima Genomics). Following the manufacturer’s instructions, tissue was fixed and DNA crosslinked using TC buffer to a final formaldehyde concentration of 2%. The tissue was homogenised using the Diagnocine Power Masher-II. Crosslinked DNA was digested with a restriction enzyme master mix, biotinylated, and ligated. Clean-up was performed with SPRISelect beads before library preparation. DNA concentration was measured with the Qubit Fluorometer (Thermo Fisher Scientific) and Qubit HS Assay Kit. The biotinylation percentage was estimated using the Arima-HiC v2 QC beads.

***Hi-C library preparation and sequencing***

Biotinylated DNA constructs were fragmented using a Covaris E220 sonicator and size selected to 400–600 bp using SPRISelect beads. DNA was enriched with Arima-HiC v2 kit Enrichment beads. End repair, A-tailing, and adapter ligation were carried out with the NEBNext Ultra II DNA Library Prep Kit (New England Biolabs), following a modified protocol where library preparation occurs while DNA remains bound to the Enrichment beads. Library amplification was performed using KAPA HiFi HotStart mix and a custom Unique Dual Index (UDI) barcode set (Integrated DNA Technologies). Depending on sample concentration and biotinylation percentage determined at the crosslinking stage, libraries were amplified with 10–16 PCR cycles. Post-PCR clean-up was performed with SPRISelect beads. Libraries were quantified using the AccuClear Ultra High Sensitivity dsDNA Standards Assay Kit (Biotium) and a FLUOstar Omega plate reader (BMG Labtech).

Prior to sequencing, libraries were normalised to 10 ng/μL. Normalised libraries were quantified again to create equimolar and/or weighted 2.8 nM pools. Pool concentrations were checked using the Agilent 4200 TapeStation (Agilent) with High Sensitivity D500 reagents before sequencing. Sequencing was performed using paired-end 150 bp reads on the Illumina NovaSeq X.

### Genome assembly

Prior to assembly of the PacBio HiFi reads, a database of *k*-mer counts ( *k* = 31) was generated from the filtered reads using FastK. GenomeScope2 ( Ranallo-Benavidez *et al.*, 2020) was used to analyse the *k*-mer frequency distributions, providing estimates of genome size, heterozygosity, and repeat content.

The HiFi reads were assembled using Hifiasm ( Cheng *et al.*, 2021) with the —primary option. The Hi-C reads ( Rao *et al.*, 2014) were mapped to the primary contigs using bwa-mem2 ( Vasimuddin *et al.*, 2019), and the contigs were scaffolded in YaHS ( Zhou *et al.*, 2023) with the —break option for handling potential misassemblies. The scaffolded assemblies were evaluated using Gfastats ( Formenti *et al.*, 2022), BUSCO ( Manni *et al.*, 2021) and MerquryFK ( Rhie *et al.*, 2020).

The mitochondrial genome was assembled using MitoHiFi ( Uliano-Silva *et al.*, 2023).

### Assembly curation

The assembly was decontaminated using the Assembly Screen for Cobionts and Contaminants ( ASCC) pipeline. TreeVal was used to generate the flat files and maps for use in curation. Manual curation was conducted primarily in PretextView and HiGlass ( Kerpedjiev *et al.*, 2018). Scaffolds were visually inspected and corrected as described by Howe *et al*. (2021). Manual corrections included 38 breaks and 43 joins. This increased the scaffold count by 5.1% and increased the total assembly length by 1.0%. The curation process is described at sanger-tol/curation-resources . PretextSnapshot was used to generate a Hi-C contact map of the final assembly.

### Assembly quality assessment

The MerquryFK tool ( Rhie *et al.*, 2020) was run in a Singularity container ( Kurtzer *et al.*, 2017) to evaluate *k*-mer completeness and assembly quality for the primary and alternate haplotypes using the *k*-mer database ( *k* = 31) computed prior to genome assembly. The analysis outputs included assembly QV scores and completeness statistics.

The genome was analysed using the BlobToolKit pipeline, a Nextflow implementation of the earlier Snakemake version ( Challis *et al.*, 2020). PacBio reads were aligned to the assembly using minimap2 ( Li, 2018) and processed with SAMtools ( Danecek *et al.*, 2021) to generate coverage tracks. The pipeline runs BUSCO ( Manni *et al.*, 2021) using lineages identified from the NCBI Taxonomy ( Schoch *et al.*, 2020). For the three basal BUSCO lineages, eukaryota, bacteria and archaea, BUSCO genes are aligned to the UniProt Reference Proteomes database ( Bateman *et al.*, 2023) using DIAMOND BLASTp ( Buchfink *et al.*, 2021). The genome assembly is divided into chunks based on the density of BUSCO genes from the closest taxonomic lineage, and each chunk is aligned to the UniProt Reference Proteomes database using DIAMOND BLASTx. Sequences without DIAMOND BLASTx hits are extracted using seqtk and aligned to the NT database using BLASTn ( Altschul *et al.*, 1990). The outputs are consolidated into a BlobDir for visualisation in BlobToolKit. The BlobToolKit pipeline was developed using nf-core tooling ( Ewels *et al.*, 2020) and MultiQC ( Ewels *et al.*, 2016), with containerisation through Docker ( Merkel, 2014) and Singularity ( Kurtzer *et al.*, 2017).

## Genome sequence report

### Sequence data

**Figure 2.**
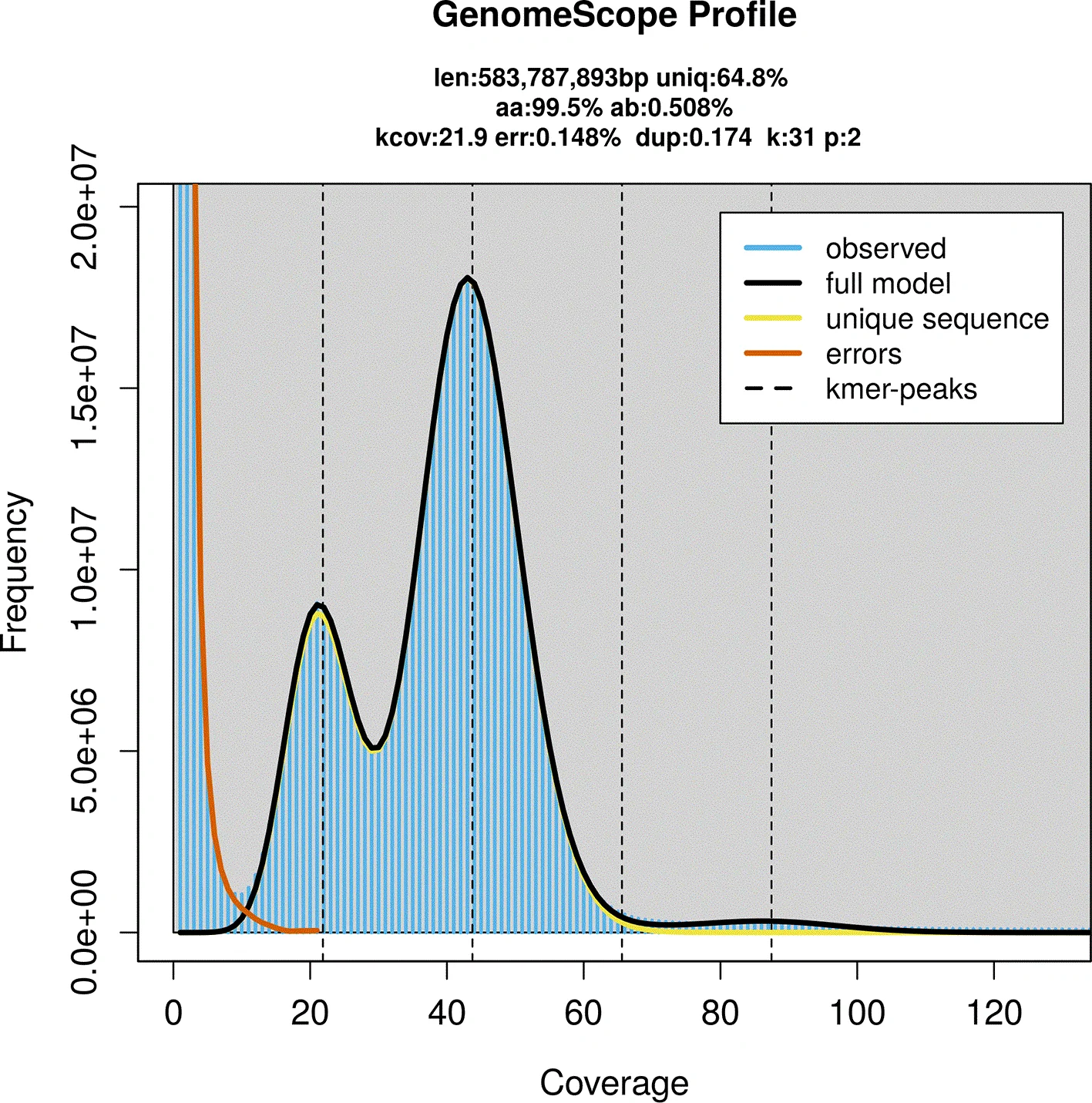
Frequency distribution of *k*-mers generated using GenomeScope2. The plot shows observed and modelled *k*-mer spectra, providing estimates of genome size, heterozygosity, and repeat content based on unassembled sequencing reads.

PacBio sequencing of the *Pseudoswammerdamia combinella* specimen generated 26.81 Gb (gigabases) from 2.59 million reads, which were used to assemble the genome. GenomeScope2.0 analysis using a *p* = 2 model gave a *k*-mer-based genome size estimate of 583.79 Mb, with a heterozygosity of 0.51% and repeat content of 35.24% ( Figure 2). These estimates guided expectations for the assembly. Based on the estimated genome size, the sequencing data provided approximately 44× coverage. Hi-C sequencing produced 122.15 Gb from 404.45 million reads, which were used to scaffold the assembly. Table 1 summarises the specimen and sequencing details.

**Table 1.** Specimen and sequencing data for *Pseudoswammerdamia combinella* (BioProject PRJEB74994).

Platform	PacBio HiFi	Hi-C
**ToLID**	ilPseComb1	ilPseComb2
**Specimen ID**	NHMUK014536857	NHMUK013697056
**BioSample (source individual)**	SAMEA112221834	SAMEA112964163
**BioSample (tissue)**	SAMEA112221925	SAMEA112975318
**Tissue**	Head, thorax and abdomen	head and thorax
**Instrument**	Sequel IIe	Illumina NovaSeq X
**Run accessions**	ERR12921329	ERR12945484
**Read count total**	2.59 million reads	404.45 million read pairs
**Base count total**	26.81 Gb	122.15 Gb

### Assembly statistics

The primary haplotype was assembled, and contigs corresponding to an alternate haplotype were also deposited in INSDC databases. The final assembly has a total length of 604.80 Mb in 61 scaffolds, with 101 gaps, and a scaffold N50 of 20.64 Mb ( Table 2).

**Table 2.** Genome assembly data for *Pseudoswammerdamia combinella.*

Genome assembly	Primary assembly
**Assembly name**	ilPseComb1.1
**Assembly accession**	GCA_965234895.1
**Alternate haplotype accession**	GCA_965234965.1
**Assembly level**	chromosome
**Span (Mb)**	604.80
**Number of chromosomes**	32
**Number of contigs**	162
**Contig N50**	7.47 Mb
**Number of scaffolds**	61
**Scaffold N50**	20.64 Mb
**Sex chromosomes**	W and Z
**Organelles**	Mitochondrial genome: 16.16 kb

**Figure 3.**
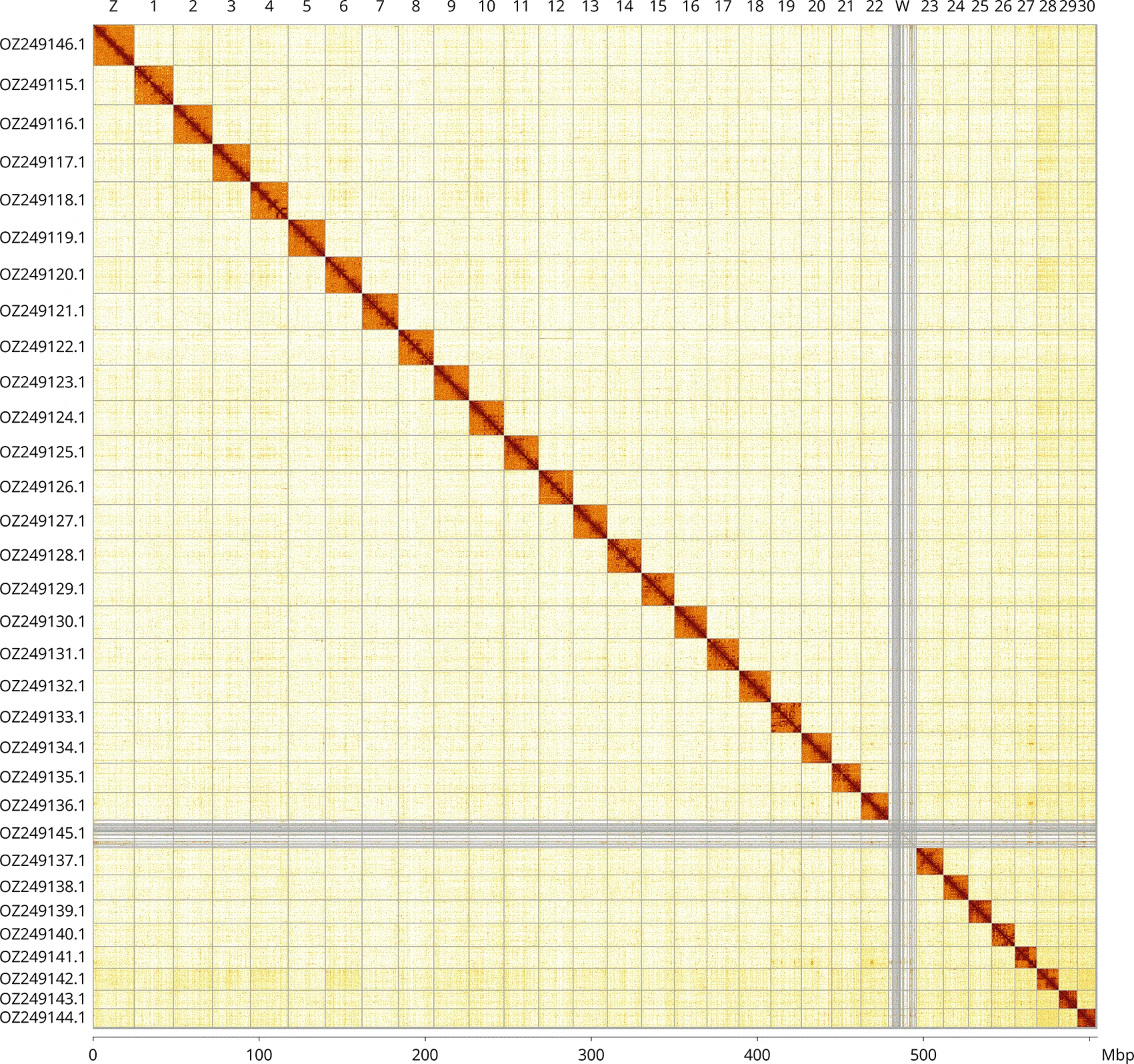
Hi-C contact map of the *Pseudoswammerdamia combinella* genome assembly. Assembled chromosomes are shown in order of size and labelled along the axes, with a megabase scale shown below. The plot was generated using PretextSnapshot.

Most of the assembly sequence (99.84%) was assigned to 32 chromosomal-level scaffolds, representing 30 autosomes and the W and Z sex chromosomes. These chromosome-level scaffolds, confirmed by Hi-C data, are named according to size ( Figure 3; Table 3). Chromosome Z and W assigned based on read coverage.

**Table 3.** Chromosomal pseudomolecules in the primary genome assembly of *Pseudoswammerdamia combinella* ilPseComb1.

INSDC accession	Molecule	Length (Mb)	GC%
OZ249115.1	1	23.61	38.50
OZ249116.1	2	23.48	39
OZ249117.1	3	22.79	39
OZ249118.1	4	22.79	39
OZ249119.1	5	22.32	38.50
OZ249120.1	6	22.18	39
OZ249121.1	7	21.74	38.50
OZ249122.1	8	21.44	38.50
OZ249123.1	9	21.23	39
OZ249124.1	10	21.07	38.50
OZ249125.1	11	20.88	39
OZ249126.1	12	20.66	38.50
OZ249127.1	13	20.64	38.50
OZ249128.1	14	20.48	38.50
OZ249129.1	15	19.94	38.50
OZ249130.1	16	19.50	38.50
OZ249131.1	17	19.41	39
OZ249132.1	18	19.12	39
OZ249133.1	19	18.40	39
OZ249134.1	20	18.23	38.50
OZ249135.1	21	17.54	39
OZ249136.1	22	16.71	39
OZ249137.1	23	16.35	38.50
OZ249138.1	24	15.05	38.50
OZ249139.1	25	14.10	39
OZ249140.1	26	14	38.50
OZ249141.1	27	13.19	39
OZ249142.1	28	13.08	40
OZ249143.1	29	11.26	38.50
OZ249144.1	30	11.14	40
OZ249145.1	W	16.59	39.50
OZ249146.1	Z	24.92	39

The mitochondrial genome was also assembled (length 16.16 kb, OZ249147.1). This sequence is included as a contig in the multifasta file of the genome submission and as a standalone record.

### Assembly quality metrics

**Figure 4.**
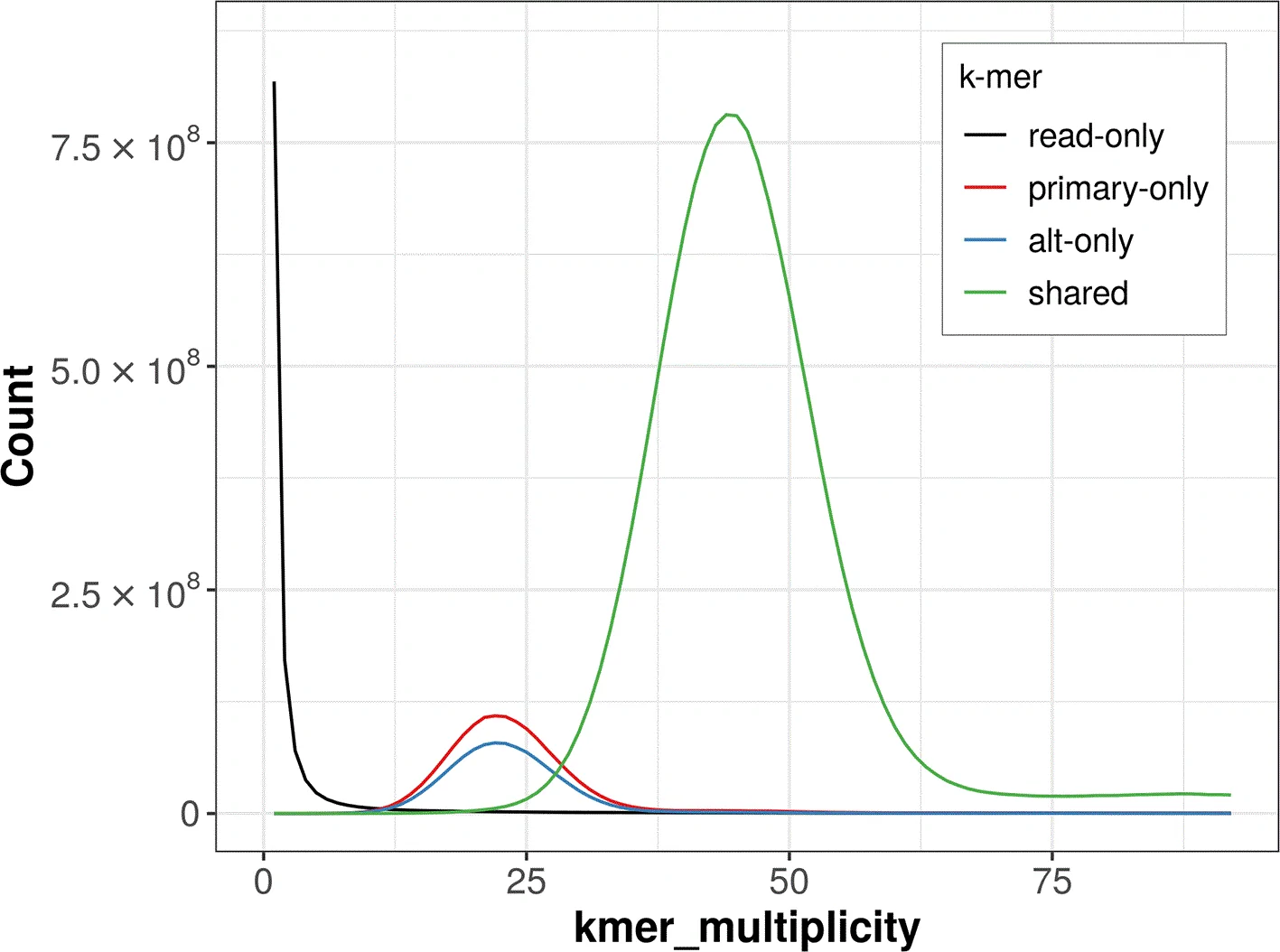
Evaluation of *k*-mer completeness using MerquryFK. This plot illustrates the recovery of *k*-mers from the original read data in the final assemblies. The horizontal axis represents *k*-mer multiplicity, and the vertical axis shows the number of *k*-mers. The black curve represents *k*-mers that appear in the reads but are not assembled. The green curve corresponds to *k*-mers shared by both haplotypes, and the red and blue curves show *k*-mers found only in one of the haplotypes.

The combined primary and alternate assemblies achieve an estimated QV of 61.5. The *k*-mer completeness is 89.28% for the primary assembly, 85.40% for the alternate haplotype, and 99.14% for the combined assemblies ( Figure 4).

**Figure 5.**
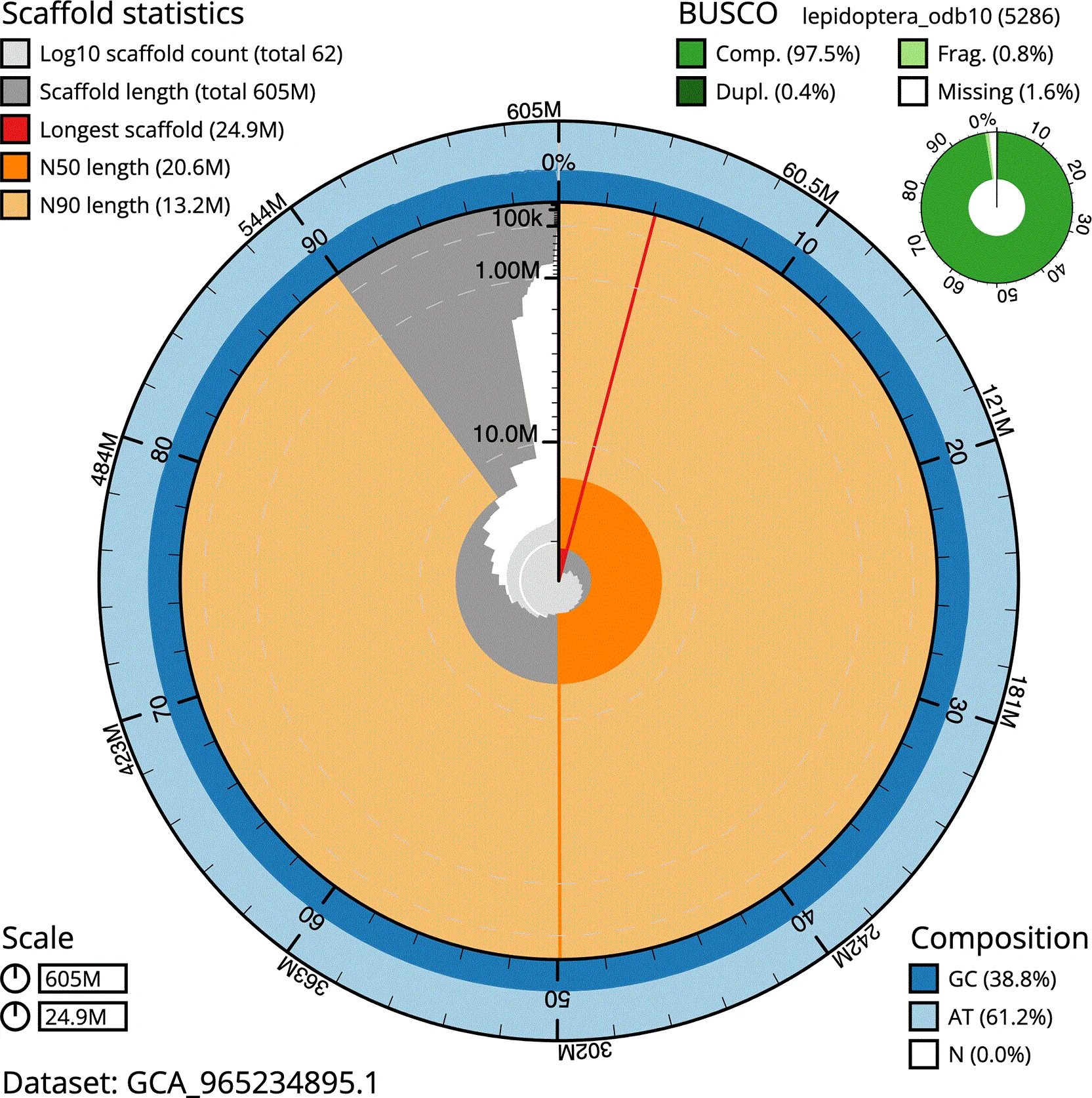
Assembly metrics for ilPseComb1.1. The BlobToolKit snail plot provides an overview of assembly metrics and BUSCO gene completeness. The circumference represents the length of the whole genome sequence, and the main plot is divided into 1 000 bins around the circumference. The outermost blue tracks display the distribution of GC, AT, and N percentages across the bins. Scaffolds are arranged clockwise from longest to shortest and are depicted in dark grey. The longest scaffold is indicated by the red arc, and the deeper orange and pale orange arcs represent the N50 and N90 lengths. A light grey spiral at the centre shows the cumulative scaffold count on a logarithmic scale. A summary of complete, fragmented, duplicated, and missing BUSCO genes in the lepidoptera_odb10 set is presented at the top right. An interactive version of this figure can be accessed on the BlobToolKit viewer.

**Figure 6.**
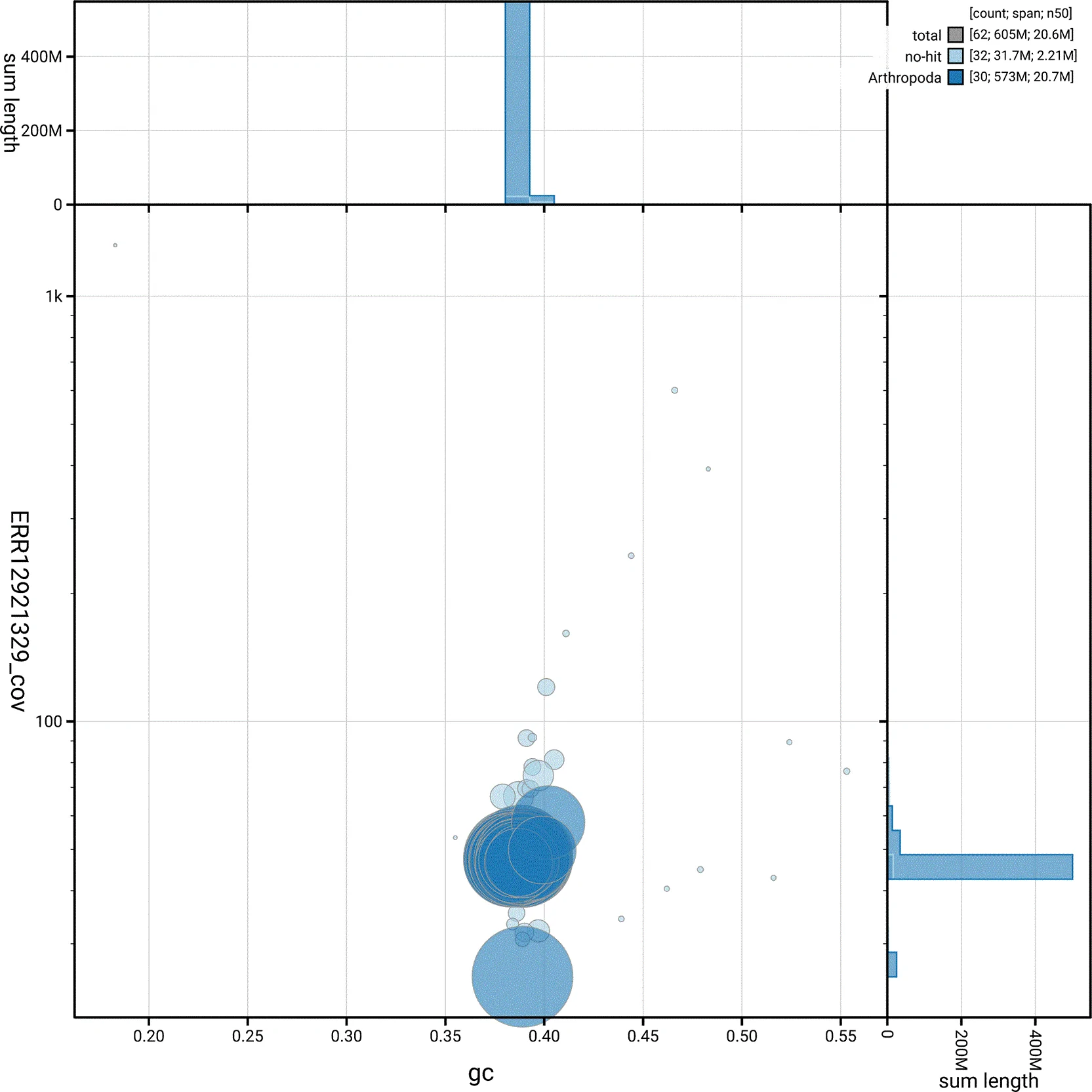
BlobToolKit blob plot for ilPseComb1.1. The plot shows base coverage (vertical axis) and GC content (horizontal axis). The circles represent scaffolds, with the size proportional to scaffold length and the colour representing phylum membership. The histograms along the axes display the total length of sequences distributed across different levels of coverage and GC content. An interactive version of this figure is available on the BlobToolKit viewer.

BUSCO v.5.7.1 analysis using the lepidoptera_odb10 reference set ( *n* = 5 286) identified 97.5% of the expected gene set (single = 97.1%, duplicated = 0.4%). The snail plot in Figure 5 summarises the scaffold length distribution and other assembly statistics for the primary assembly. The blob plot in Figure 6 shows the distribution of scaffolds by GC proportion and coverage.

Table 4 lists the assembly metric benchmarks adapted from Rhie *et al.* (2021) and the Earth BioGenome Project Report on Assembly Standards. The EBP metric, calculated for primary assembly, is **6.C.Q62**, meeting the recommended 6.C.Q40 reference standard.

**Table 4.** Earth Biogenome Project summary metrics for the *Pseudoswammerdamia combinella* assembly.

Measure	Value	Benchmark
EBP summary (primary)	6.C.Q62	6.C.Q40
Contig N50 length	7.47 Mb	≥ 1 Mb
Scaffold N50 length	20.64 Mb	= chromosome N50
Consensus quality (QV)	Primary: 62.2; alternate: 61.2; combined: 61.5	≥ 40
*k*-mer completeness	Primary: 89.28%; alternate: 85.40%; combined: 99.14%	≥ 90%
BUSCO	C:97.5% [S:97.1%, D:0.4%], F:0.8%, M:1.6%, n:5 286	S > 90%; D < 5%
Percentage of assembly assigned to chromosomes	99.84%	≥ 90%

*Notes:* The EBP summary uses log10(Contig N50); chromosome-level (C) or log10(Scaffold N50); Q (Merqury QV). BUSCO: C = complete; S = single-copy; D = duplicated; F = fragmented; M = missing; n = orthologues.

**Table 5.** Software versions and sources used for *Pseudoswammerdamia combinella.*

Software	Version	Source
BLAST	2.14.0	https://ftp.ncbi.nlm.nih.gov/blast/executables/blast+/
BlobToolKit	4.4.5	https://github.com/blobtoolkit/blobtoolkit
BUSCO	5.7.1	https://gitlab.com/ezlab/busco
bwa-mem2	2.2.1	https://github.com/bwa-mem2/bwa-mem2
DIAMOND	2.1.8	https://github.com/bbuchfink/diamond
fasta_windows	0.2.4	https://github.com/tolkit/fasta_windows
FastK	1.1	https://github.com/thegenemyers/FASTK
GenomeScope2.0	2.0.1	https://github.com/tbenavi1/genomescope2.0
Gfastats	1.3.6	https://github.com/vgl-hub/gfastats
Hifiasm	0.19.8-r603	https://github.com/chhylp123/hifiasm
HiGlass	1.13.4	https://github.com/higlass/higlass
MerquryFK	1.1.0-c1	https://github.com/thegenemyers/MERQURY.FK
Minimap2	2.28-r1209	https://github.com/lh3/minimap2
MitoHiFi	3	https://github.com/marcelauliano/MitoHiFi
MultiQC	1.14; 1.17 and 1.18	https://github.com/MultiQC/MultiQC
Nextflow	24.10.4	https://github.com/nextflow-io/nextflow
PretextSnapshot	0.0.5	https://github.com/sanger-tol/PretextSnapshot
PretextView	1.0.3	https://github.com/sanger-tol/PretextView
samtools	1.21	https://github.com/samtools/samtools
sanger-tol/ascc	0.1.0	https://github.com/sanger-tol/ascc
sanger-tol/blobtoolkit	v0.7.1	https://github.com/sanger-tol/blobtoolkit
sanger-tol/curationpretext	1.4.2	https://github.com/sanger-tol/curationpretext
Seqtk	1.3	https://github.com/lh3/seqtk
Singularity	3.9.0	https://github.com/sylabs/singularity
TreeVal	1.4.0	https://github.com/sanger-tol/treeval
YaHS	1.2a.2	https://github.com/c-zhou/yahs

## Author information

Contributors are listed at the following links:

• Members of the Natural History Museum Genome Acquisition Lab
• Members of the Darwin Tree of Life Barcoding collective
• Members of the Wellcome Sanger Institute Tree of Life Management, Samples and Laboratory team
• Members of Wellcome Sanger Institute Scientific Operations – Sequencing Operations
• Members of the Wellcome Sanger Institute Tree of Life Core Informatics team
• Members of the Tree of Life Core Informatics collective
• Members of the Darwin Tree of Life Consortium

## Wellcome Sanger Institute – Legal and Governance

The materials that have contributed to this genome note have been supplied by a Darwin Tree of Life Partner. The submission of materials by a Darwin Tree of Life Partner is subject to the **‘Darwin Tree of Life Project Sampling Code of Practice’**, which can be found in full on the Darwin Tree of Life website. By agreeing with and signing up to the Sampling Code of Practice, the Darwin Tree of Life Partner agrees they will meet the legal and ethical requirements and standards set out within this document in respect of all samples acquired for, and supplied to, the Darwin Tree of Life Project. Further, the Wellcome Sanger Institute employs a process whereby due diligence is carried out proportionate to the nature of the materials themselves, and the circumstances under which they have been/are to be collected and provided for use. The purpose of this is to address and mitigate any potential legal and/or ethical implications of receipt and use of the materials as part of the research project, and to ensure that in doing so we align with best practice wherever possible. The overarching areas of consideration are:

• Ethical review of provenance and sourcing of the material
• Legality of collection, transfer and use (national and international)

Each transfer of samples is further undertaken according to a Research Collaboration Agreement or Material Transfer Agreement entered into by the Darwin Tree of Life Partner, Genome Research Limited (operating as the Wellcome Sanger Institute), and in some circumstances, other Darwin Tree of Life collaborators.

## Data Availability

European Nucleotide Archive: Pseudoswammerdamia combinella. Accession number PRJEB74994; https://identifiers.org/ena.embl/PRJEB74994. The genome sequence is released openly for reuse. The *Pseudoswammerdamia combinella* genome sequencing initiative is part of the Darwin Tree of Life Project (PRJEB40665), the Sanger Institute Tree of Life Programme (PRJEB43745) and Project Psyche (PRJEB71705). All raw sequence data and the assembly have been deposited in INSDC databases. The genome will be annotated using available RNA-Seq data and presented through the Ensembl pipeline at the European Bioinformatics Institute. Raw data and assembly accession identifiers are reported in Tables 1 and 2. Production code used in genome assembly at the WSI Tree of Life is available at https://github.com/sanger-tol . Table 5 lists software versions used in this study.

## References

[ref2] Altschul SF Gish W Miller W : Basic Local Alignment Search Tool. *J. Mol. Biol.* 1990;215(3):403–410. 10.1016/S0022-2836(05)80360-2 2231712

[ref3] Bateman A Martin M-J Orchard S : UniProt: The Universal Protein Knowledgebase in 2023. *Nucleic Acids Res.* 2023;51(D1):D523–D531. 10.1093/nar/gkac1052 36408920 PMC9825514

[ref4] Buchfink B Reuter K Drost H-G : Sensitive protein alignments at tree-of-life scale using DIAMOND. *Nat. Methods.* 2021;18(4):366–368. 10.1038/s41592-021-01101-x 33828273 PMC8026399

[ref5] Challis R Richards E Rajan J : BlobToolKit – interactive quality assessment of genome assemblies. *G3: Genes, Genomes Genetics.* 2020;10(4):1361–1374. 10.1534/g3.119.400908 32071071 PMC7144090

[ref6] Cheng H Concepcion GT Feng X : Haplotype-resolved *de novo.* assembly using phased assembly graphs with Hifiasm. *Nat. Methods.* 2021;18(2):170–175. 10.1038/s41592-020-01056-5 33526886 PMC7961889

[ref7] Crowley L Allen H Barnes I : A sampling strategy for genome sequencing the British terrestrial Arthropod fauna. *Wellcome Open Research.* 2023;8:123. 10.12688/wellcomeopenres.18925.1 37408610 PMC10318377

[ref8] Danecek P Bonfield JK Liddle J : Twelve years of SAMtools and BCFtools. *GigaScience.* 2021;10(2):giab008. 10.1093/gigascience/giab008 33590861 PMC7931819

[ref59] Davis T : A review of the status of microlepidoptera. 2012. Reference source

[ref9] Ewels P Magnusson M Lundin S : MultiQC: Summarize analysis results for multiple tools and samples in a single report. *Bioinformatics.* 2016;32(19):3047–48. 10.1093/bioinformatics/btw354 27312411 PMC5039924

[ref10] Ewels PA Peltzer A Fillinger S : The nf-core framework for community-curated bioinformatics pipelines. *Nat. Biotechnol.* 2020;38(3):276–278. 10.1038/s41587-020-0439-x 32055031

[ref11] Formenti G Abueg L Brajuka A : Gfastats: Conversion, evaluation and manipulation of genome sequences using assembly graphs. *Bioinformatics.* 2022;38(17):4214–4216. 10.1093/bioinformatics/btac460 35799367 PMC9438950

[ref12] GBIF Secretariat: *Pseudoswammerdamia combinella.* (Hübner, 1786). *GBIF Backbone Taxonomy.* 2023. 10.15468/39omei Reference source

[ref13] Howard C Denton A Jackson B : On the path to reference genomes for all biodiversity: Lessons learned and laboratory protocols created in the Sanger Tree of Life core laboratory over the first 2000 species. *bioRxiv.* 2025. 10.1101/2025.04.11.648334 PMC12548527 41129326

[ref14] Howe K Chow W Collins J : Significantly improving the quality of genome assemblies through curation. *GigaScience.* 2021;10(1):giaa153. 10.1093/gigascience/giaa153 33420778 PMC7794651

[ref15] Kerpedjiev P Abdennur N Lekschas F : HiGlass: Web-based visual exploration and analysis of genome interaction maps. *Genome Biol.* 2018;19(1):125. 10.1186/s13059-018-1486-1 30143029 PMC6109259

[ref16] Kurtzer GM Sochat V Bauer MW : Singularity: Scientific containers for mobility of compute. *PLOS ONE.* 2017;12(5):e0177459. 10.1371/journal.pone.0177459 28494014 PMC5426675

[ref17] Li H : Minimap2: Pairwise alignment for nucleotide sequences. *Bioinformatics.* 2018;34(18):3094–3100. 10.1093/bioinformatics/bty191 29750242 PMC6137996

[ref18] Manni M Berkeley MR Seppey M : BUSCO update: Novel and streamlined workflows along with broader and deeper phylogenetic coverage for scoring of eukaryotic, prokaryotic, and viral genomes. *Mol. Biol. Evol.* 2021;38(10):4647–4654. 10.1093/molbev/msab199 34320186 PMC8476166

[ref19] Merkel D : Docker: Lightweight Linux containers for consistent development and deployment. *Linux J.* 2014;2014(239):2. 10.5555/2600239.2600241

[ref20] Ranallo-Benavidez TR Jaron KS Schatz MC : GenomeScope 2.0 and Smudgeplot for reference-free profiling of polyploid genomes. *Nat. Commun.* 2020;11(1):1432. 10.1038/s41467-020-14998-3 32188846 PMC7080791

[ref21] Rao SSP Huntley MH Durand NC : A 3D map of the human genome at kilobase resolution reveals principles of chromatin looping. *Cell.* 2014;159(7):1665–1680. 10.1016/j.cell.2014.11.021 25497547 PMC5635824

[ref22] Rhie A McCarthy SA Fedrigo O : Towards complete and error-free genome assemblies of all vertebrate species. *Nature.* 2021;592(7856):737–746. 10.1038/s41586-021-03451-0 33911273 PMC8081667

[ref23] Rhie A Walenz BP Koren S : Merqury: Reference-free quality, completeness, and phasing assessment for genome assemblies. *Genome Biol.* 2020;21(1):245. 10.1186/s13059-020-02134-9PMID: PMCID: 32928274 PMC7488777

[ref24] Schoch CL Ciufo S Domrachev M : NCBI taxonomy: A comprehensive update on curation, resources and tools. *Database.* 2020;2020:baaa062. 10.1093/database/baaa062, PMID: , PMCID: 32761142 PMC7408187

[ref25] Sterling P Parsons M Lewington R : *Field Guide to the Micro-Moths of Great Britain and Ireland.* Gillingham, Dorset, UK: British Wildlife Publishing;2012.

[ref26] Twyford AD Beasley J Barnes I : A DNA barcoding framework for taxonomic verification in the Darwin Tree of Life Project. *Wellcome Open Research.* 2024;9:339. 10.12688/wellcomeopenres.21143.1 39386966 PMC11462125

[ref27] Uliano-Silva M Ferreira JGRN Krasheninnikova K : MitoHiFi: A Python pipeline for mitochondrial genome assembly from PacBio high fidelity reads. *BMC Bioinformatics.* 2023;24(1):288. 10.1186/s12859-023-05385-y 37464285 PMC10354987

[ref28] Vasimuddin M Misra S Li H : Efficient architecture-aware acceleration of BWA-MEM for multicore systems. *IEEE International Parallel and Distributed Processing Symposium (IPDPS).* IEEE;2019;2019:314–24. 10.1109/IPDPS.2019.00041

[ref29] Zhou C McCarthy SA Durbin R : YaHS: Yet another Hi-C scaffolding tool. *Bioinformatics.* 2023;39(1):btac808. 10.1093/bioinformatics/btac808 36525368 PMC9848053

