# Supplementary material for: The genome sequence of the Copper-tipped Ermine, Pseudoswammerdamia combinella (Hübner, 1786) (Lepidoptera: Yponomeutidae)
Source: Wellcome Open Res. 2026 Aug 21;11:537. [Version 1] doi: 10.12688/wellcomeopenres.27367.1 (PMC13583344; doi:10.12688/wellcomeopenres.27367.1)
Supplement: Peer review history

# Reviewer report: Reviewer response for version 1

**DOI:** 10.21956/wellcomeopenres.30122.r165529

**Published:** 2026-09-16

**Authors:** Isaac Hinne

**Affiliations:** 1 University of Nevada Reno, Reno, Nevada, USA

The authors present a 604.80 Mb chromosome-level genome assembly for the Copper-tipped Ermine with 94% of the sequence assigned to 32 chromosomal pseudomolecules, including the W and Z chromosomes. The genome is highly complete, as inferred by a 97.5% BUSCO completeness and a primary consensus quality of QV 62.2. The methods used are sound and follow standard Darwin ToL protocols.

 My main concern is with the following points:

 The manuscript states that the W and Z chromosomes were assigned based on read coverage, but it does not report their coverage relative to the autosomes or explain how the sex chromosomes were distinguished. In a female W individual, both sex chromosomes are expected to have reduced coverage relative to diploid autosomes. Therefore, coverage alone does not establish which scaffold represents either W or Z. Please report the observed coverage and explain any additional evidence used.

 Table 2 and the results report 61 scaffolds, whereas Figure 5 shows 62. You may have included the mitochondrial sequence here, but you should state that.

 Please correct the malformed citation in the third line of the background. 

 Table 1 reports 404.45 million HiC-read pairs, whereas the result describes the same quantity as reads. Please reconcile.

Are sufficient details of methods and materials provided to allow replication by others?

Yes

Is the rationale for creating the dataset(s) clearly described?

Yes

Are the datasets clearly presented in a useable and accessible format?

Yes

Are the protocols appropriate and is the work technically sound?

Yes

Reviewer Expertise:

Arthropod genomics

I confirm that I have read this submission and believe that I have an appropriate level of expertise to confirm that it is of an acceptable scientific standard.

# Reviewer report: Reviewer response for version 1

**DOI:** 10.21956/wellcomeopenres.30122.r167503

**Published:** 2026-09-16

**Authors:** Matthew Merkin

**Affiliations:** 1 University of Liverpool, Liverpool, UK

The authors present a high quality reference genome for *Pseudoswammerdamia combinella *as part of the Darwin Tree of Life (DToL) project. The background is of exceptional quality for a genome note, succinctly providing relevant species information and why it was sequenced. They extracted DNA from two samples and, after confirming the species by barcoding, performed HiC and HiFi sequencing. The standard DToL pipeline was then used for genome assembly and QC. The summary metrics in table 4 are all greater than the benchmark values, indicating that this genome assembly is of high quality and suitable to be used as a reference.

 However, this genome note currently lacks an annotation report. There is a commitment in the Data availability to annotate the genome later on, but these genome notes greatly benefit from discussion of the annotation, so its indexing without the annotation seems premature. 

 It is also interesting to see a mitochondrial genome greater than 16kb in Lepidoptera (although it is only marginally larger in this case). It would be good to add an additional sentence explaining why, such as through a gene duplication or expansion of the non-coding region.

Are sufficient details of methods and materials provided to allow replication by others?

Yes

Is the rationale for creating the dataset(s) clearly described?

Yes

Are the datasets clearly presented in a useable and accessible format?

Yes

Are the protocols appropriate and is the work technically sound?

Yes

Reviewer Expertise:

population genomics, museomics, Lepidoptera

I confirm that I have read this submission and believe that I have an appropriate level of expertise to confirm that it is of an acceptable scientific standard.

# Reviewer report: Reviewer response for version 1

**DOI:** 10.21956/wellcomeopenres.30122.r165530

**Published:** 2026-09-09

**Authors:** Anwesha Das

**Affiliations:** 1 Sanaka Educational Trust’s Group of Institutions (SETGOI), Durgapur, West Bengal, India

Overall assessment

 This Data Note presents a chromosome-level genome assembly for *Pseudoswammerdamia combinella*, a relatively understudied microlepidopteran species, generated as part of the Darwin Tree of Life project. The study provides a valuable genomic resource, with a 604.80 Mb assembly, 32 chromosomal pseudomolecules representing 30 autosomes and the W and Z sex chromosomes, and a mitochondrial genome of 16.16 kb. The reported assembly quality is generally strong, including a primary assembly consensus QV of 62.2, 97.5% complete BUSCOs, and 99.84% of the assembly assigned to chromosomes.

 The experimental and computational workflow is clearly described and uses established sequencing, assembly, scaffolding and quality-assessment approaches. The open deposition of raw sequencing data and genome assemblies further increases the utility of this resource.

 I consider the manuscript potentially suitable for indexing after **minor revision**. The principal areas that would benefit from clarification concern the discrepancy between the estimated genome size and final assembly size, interpretation of k-mer completeness, chromosome and sex-chromosome assignment, sequencing/QC information, and details of manual curation. Addressing these points would improve transparency and reproducibility without requiring substantial additional experimental work.

**Major comments**

 1. Clarification of the GenomeScope2 genome-size estimate versus final assembly size

 GenomeScope2 estimated the genome size at 583.79 Mb, whereas the final primary assembly is 604.80 Mb, approximately 3.6% larger. The manuscript currently reports both values but does not explain the difference.

 Please provide a brief explanation of why the final assembly exceeds the k-mer-based genome-size estimate. Possible contributing factors could include repeat-rich or low-copy sequences, limitations of the GenomeScope model, heterozygosity, assembly of sequences not fully represented in the dominant k-mer spectrum, or the inclusion of sequences associated with the sex chromosomes. The authors should clarify whether the difference is considered within the expected range for this type of estimate.

 2. More detail on sequencing-read filtering and quality control

 The Methods state that a k-mer database was generated from “filtered reads”, but the criteria used for filtering are not described.

 Please specify:

- the filtering software and version;
- quality or length thresholds, if applied;
- whether adapter removal was performed;
- the number or proportion of reads/bases retained after filtering; and
- whether any reads were excluded before Hifiasm assembly.

This information would make the sequencing-to-assembly workflow more reproducible.

 3. Evidence supporting the chromosome number and sex-chromosome assignments

 The manuscript reports 32 chromosomes, consisting of 30 autosomes plus W and Z, and states that chromosome-level scaffolds were confirmed by Hi-C data. However, the W and Z chromosomes were assigned based on read coverage.

 Please provide a little more detail regarding the evidence used to distinguish the W and Z chromosomes from autosomes. In particular, it would be helpful to state whether the assignment was based solely on relative PacBio coverage or whether other characteristics, such as Hi-C interaction patterns, sequence composition, known synteny, or comparison with related Lepidoptera, were considered.

 Because sex chromosomes can have substantially different coverage and repetitive content, a short explanation of the assignment criteria would strengthen this aspect of the assembly.

 4. Interpretation of the scaffold N50 in the context of chromosome-level assembly

 Although 99.84% of the assembly is assigned to 32 chromosome-level pseudomolecules, the reported scaffold N50 is 20.64 Mb, compared with a total assembly length of 604.80 Mb.

 Please clarify how the remaining sequence is distributed among unplaced or smaller scaffolds and whether these sequences are expected to represent unresolved regions, repetitive sequences, organellar/contaminant sequences, or genuinely unplaced nuclear sequence. It would also be useful to indicate the amount of sequence that remains outside the 32 chromosomal pseudomolecules.

 5. Interpretation of k-mer completeness

 The primary assembly has a k-mer completeness of 89.28%, whereas the combined primary and alternate assemblies achieve 99.14%.

 This is an important point because the combined value exceeds the stated EBP benchmark, but the primary assembly alone falls below 90%. Please explain the biological and assembly-related reasons for this difference and clarify how the authors interpret the 89.28% completeness of the primary reference assembly.

 In particular, the authors should discuss whether the missing k-mers are predominantly associated with heterozygous sequence, repetitive regions, or other components represented in the alternate haplotype. A brief interpretation would help readers understand the completeness of the primary reference genome.

 6. Description and intended use of the alternate haplotype

 The manuscript states that contigs corresponding to an alternate haplotype were also deposited in INSDC databases, but relatively little information is provided about its structure and generation.

 Please clarify:

- how the alternate haplotype was generated from the Hifiasm assembly;
- whether it is fully phased or represents a collection of alternate contigs;
- its total length and contig/scaffold statistics, if available; and
- how users should interpret or use the alternate assembly.

This is particularly relevant given the substantial contribution of the alternate haplotype to the combined k-mer completeness.

**Minor comments**

 7. Correct the apparent taxonomic/common-name typo

 In the Background, the species is described as being known as the “Copper-tipped Ermel”. This appears inconsistent with the manuscript title and the more commonly used name “Copper-tipped Ermine”.

 Please check and correct this terminology if it is a typographical error.

 8. Clarify the statement concerning geographic distribution

 The manuscript states that the species occurs across Britain but is more sparsely distributed in Scotland and Northern Ireland and subsequently describes a wider Palaearctic distribution.

 The geographic description could be made slightly more precise, preferably by distinguishing the British/Irish distribution from the broader European/Palaearctic range and ensuring that the cited sources directly support each statement.

 9. Rationale for using two specimens

 Two female specimens were used, with one providing PacBio HiFi data and the other providing Hi-C data.

 Please briefly explain the rationale for using different individuals for the genome and Hi-C datasets. Since Hi-C scaffolding was performed using a specimen distinct from the individual used for genome sequencing, it would be useful to comment on whether potential individual-level variation could affect scaffolding or haplotype structure.

 10. Report sequencing depth consistently

 The manuscript states that 26.81 Gb of PacBio data corresponded to approximately “44 coverage”.

 Please revise this to “approximately 44× coverage” and, if possible, report the effective coverage after filtering rather than only the theoretical coverage calculated from the GenomeScope genome-size estimate.

 11. Provide additional Hi-C quality metrics

 The manuscript provides the total Hi-C sequencing yield (122.15 Gb from 404.45 million read pairs), but no mapping rate, valid-pair rate, duplicate rate, or proportion of informative Hi-C contacts is reported.

 If these metrics are available, including them would provide useful evidence supporting the quality of the chromosome-scale scaffolding.

 12. Expand the description of manual curation

 The authors report 38 breaks and 43 joins during manual curation, which increased scaffold count by 5.1% and assembly length by 1.0%.

 Please provide a brief explanation of the criteria used to identify misassemblies and make these corrections. For example, were corrections based primarily on abnormal Hi-C contact patterns, read coverage, assembly graphs, or combinations of these evidence types?

 13. Clarify contamination assessment

 The Assembly Screen for Cobionts and Contaminants (ASCC) pipeline was used for decontamination, and BlobToolKit was subsequently used to assess taxonomic composition.

 It would strengthen the report to provide a brief quantitative statement regarding whether any contaminant sequences were detected and removed, and the approximate amount of sequence affected, if applicable.

 14. Provide mitochondrial genome quality information

 The mitochondrial genome is reported as 16.16 kb and deposited as accession OZ249147.1.

 If available from the MitoHiFi analysis, please report whether the mitochondrial genome is complete and circular and whether expected mitochondrial genes were identified. This would provide a more complete assessment of the organellar assembly.

 15. Consolidate accession information

 The Data Availability section provides the BioProject accession and refers readers to Tables 1 and 2 for raw-data and assembly identifiers.

 For ease of reuse, consider presenting the key BioProject, BioSample, raw-read, primary assembly, alternate haplotype and mitochondrial accessions together in one concise list or table.

 16. Moderate some statements regarding downstream applications

 The Background states that the assembly “supports comparative and functional genomic research into insect neurobiology, the development of the visual system, and specialisation on host plants”.

 Given that the present study is a genome assembly/data note without functional genomic analyses, this could be phrased more cautiously—for example, that the genome “may facilitate” or “provides a resource for” comparative and functional genomic studies in these areas.

 17. Minor editorial and typographical corrections

 A final editorial pass would improve presentation. In addition to the “Copper-tipped Ermel” issue noted above, please check:

- consistency in use of *P. combinella* versus the full species name;
- formatting of sequencing coverage as “44×”;
- consistency in the presentation of units (Mb, kb, Gb);
- consistency between text and tables for assembly statistics; and
- formatting and completeness of software/database URLs and accession identifiers.

Are sufficient details of methods and materials provided to allow replication by others?

Partly

Is the rationale for creating the dataset(s) clearly described?

Partly

Are the datasets clearly presented in a useable and accessible format?

Partly

Are the protocols appropriate and is the work technically sound?

Partly

Reviewer Expertise:

Computational and medicinal chemistry

I confirm that I have read this submission and believe that I have an appropriate level of expertise to confirm that it is of an acceptable scientific standard, however I have significant reservations, as outlined above.
